# Evaluation of basophil activation caused by transgenic rice seeds expressing whole T cell epitopes of the major Japanese cedar pollen allergens

**DOI:** 10.1186/s13601-019-0249-8

**Published:** 2019-02-20

**Authors:** Shinya Takaishi, Saburo Saito, Minori Kamada, Nobuyoshi Otori, Hiromi Kojima, Kenjiro Ozawa, Fumio Takaiwa

**Affiliations:** 1 Department of Otorhinolaryngology, Jikei University School of Medicine, Tokyo, Japan; 2 Division of Molecular Immunology, Research Center for Medical Sciences, Jikei University School of Medicine, 3-25-8 Nishi-shinbashi, Minato-ku, Tokyo, 105-8461 Japan; 3 Core Research Facilities for Basic Science (Division of Molecular Genetics), Research Center for Medical Sciences, Jikei University School of Medicine, Tokyo, Japan; 4 Core Research Facilities for Basic Science (Division of Molecular Cell Biology), Research Center for Medical Sciences, Jikei University School of Medicine, Tokyo, Japan; 5 Plant Molecular Farming Unit, Division of Biotechnology, Institute of Agrobiological Sciences, National Agriculture and Food Research Organization, Ibaraki, Japan

**Keywords:** Pollinosis, Immunotherapy, T-cell epitope, Transgenic rice, Basophil activation test

## Abstract

**Background:**

Japanese cedar (JC) pollinosis is a serious type I allergic disease in Japan. Although subcutaneous immunotherapy and sublingual immunotherapy have been applied to treat JC pollinosis, high doses of allergens may cause IgE-mediated allergic reactions. The transgenic rice seeds that contain genetically modified Cry j 1 and Cry j 2, the two major allergens of JC pollen, have been developed as candidates for oral immunotherapy. Although the antigens in the transgenic rice seeds (Tg-rice seeds) were engineered such that they decrease binding ability with IgE and they are of insufficient length to cross-link IgE on the surface of mast cells or basophils, the safety of Tg-rice seeds for patients with JC pollinosis was unclear.

**Methods:**

To verify the safety of Tg-rice seeds in terms of allergies, we investigated the percentage of activated basophils induced by Tg-rice seed extract in the basophil activation test. Blood samples from 29 patients with JC pollinosis were collected. Tg-rice seed extract, non-transgenic wild-type rice seed extract, and Cry j 1 and Cry j 2 were mixed with the blood with reagents. The percentage of activated basophils was assessed by CD203c expression, a basophil activation marker.

**Results:**

The percentage of activated basophils after the stimulation with Tg-rice seed extract was 4.5 ± 1.6% (mean ± SD) compared with 62.9 ± 20.2% after Cry j 1- and Cry j 2-stimulation (difference 58.4%, P < 0.001, 95% confidence interval 51.0–65.9%).

**Conclusions:**

The results will contribute to the safety of Tg-rice seeds in terms of allergies.

## Background

Japanese cedar pollinosis is a seasonal allergic rhinitis caused by Japanese cedar (*Cryptomeria japonica*) pollen and is one of the most serious type I allergic diseases in Japan. Japanese cedar (JC) pollinosis causes clinical symptoms of conjunctivitis and asthma, in addition to allergic rhinitis, from February to April each year, and is considered a national affliction. Patients with JC pollinosis wear facemasks and eyeglasses between February and April to prevent exposure to JC pollen. An epidemiological study conducted in 2008 indicated that the prevalence of JC pollinosis was 26.5%, representing a higher than 10% increase over the previous 10 years [1]. The reason why JC pollinosis became a common disease in the past half-century is the increased number of cedar pollens owing to global climate change and forest growth caused by the tree-planting program of the Japanese government after World War II [2]. Conventional therapies for JC pollinosis include allergen avoidance, pharmacotherapy using antihistamines or nasal steroids, and allergen-specific immunotherapy [3]. Allergen-specific immunotherapy is the only current treatment that can change the natural course of JC pollinosis with long-term effects. Subcutaneous immunotherapy (SCIT) has been widely applied as immunotherapy for JC pollinosis. Sublingual immunotherapy (SLIT) was also introduced to treat JC pollinosis in 2014 [4]. However, adverse events are associated with SCIT and even SLIT. SCIT often induces severe adverse reactions like local allergic reactions, urticaria, asthma, and anaphylaxis. One fatal reaction has been known to occur for every 2.5 million injections [5]. Predominant adverse reactions to SLIT have been mild local reactions such as oral pruritus, edema of the mouth, throat irritation, and sneezing [4, 6]. However, a few cases of anaphylaxis have also been reported after SLIT using a crude or standardized vaccine [7–11]. In both immunotherapies, high doses of allergens may cause various adverse events, including an anaphylactic reaction.

Recently, two types of transgenic rice seeds have attracted increasing attention as immunotherapeutic candidates for JC pollinosis: one contains a hybrid peptide called ‘7Crp peptide’ consisting of seven linked dominant human T-cell epitopes derived from Cry j 1 and Cry j 2, the two major allergens of JC pollen [12–14], and the other was engineered to express structurally disrupted allergens containing the whole amino acid sequences of Cry j 1 and Cry j 2, targeting all the JC pollen allergen-specific T-cells [15–17]. The development of the 7Crp peptide preceded the latter transgenic rice seed, and the 7Crp peptide has attracted attention as a peptide-based immunotherapy [18–20]. A clinical trial of the 7Crp peptide is under way in the Department of Otorhinolaryngology, Jikei University School of Medicine, Tokyo, Japan, and the rice containing 7Crp peptide is being taken orally as a seed-based peptide vaccine.

By contrast, the transgenic rice seeds expressing whole T-cell epitopes of the major JC pollen allergens Cry j 1 and Cry j 2 (Tg-rice seeds) may be used to treat a wider range of patients with different genetic backgrounds. Tg-rice seeds contain all possible T-cell epitope repertoires of the Cry j 1 and Cry j 2 antigens in the edible portion (endosperm): the Cry j 1 gene was divided into three overlapping fragments and the Cry j 2 gene was deconstructed by shuffling [15–17]. Recombinant proteins deposited in Tg-rice seeds are stable for at least 10 months at room temperature under sealed conditions [16]. Antigen is generally degraded in the gastrointestinal tract before arrival at the mucosal immune cells in gut-associated lymphoid tissue due to exposure to the harsh environment in the stomach (low PH, pepsin) [14]. However, the recombinant proteins in protein bodies in the endosperm showed a greater resistance to pepsin [16]. When orally delivered via cereal seeds such as rice grains, bio-encapsulation of antigen by the double barriers of protein bodies and the cell walls characteristic of plant cells has the advantage of protecting the antigen from proteolysis [14]. Furthermore, although rice is usually boiled before consumption, Tg-rice seeds retain antigenicity even after boiling at 100 °C for 100 min. Tg-rice seeds offer a suitable delivery medium to gut-associated lymphoid tissue due to their high stability at room temperature, ample and stable deposition space, high expression level, and protection from digestive enzymes in gut [17]. Mice fed Tg-rice seeds daily for 3 weeks and then challenged with crude JC pollen allergen showed marked suppression of allergen-specific CD4^+^ T-cell proliferation, IgE and IgG levels compared with mice fed non-transgenic rice seeds [15]. Sneezing frequency and infiltration of inflammatory cells such as eosinophils and neutrophils were also significantly reduced in the nasal tissue [15]. These results suggest that oral administration of Tg-rice seeds actually induces immune tolerance against JC pollinosis. Although antigenicity of Tg-rice seeds has been demonstrated in several studies [15–17, 21], the safety of Tg-rice seeds for patients with JC pollinosis is unclear. The antigens in Tg-rice seeds were engineered such that they decrease binding ability with immunoglobulin E (IgE) and they are of insufficient length to cross-link IgE on the surface of mast cells or basophils, which theoretically, would decrease the risk of IgE-mediated adverse events. However, the IgE-binding and IgE-crosslinking abilities of the antigens in Tg-rice seeds have not yet been verified.

We thought that these abilities could be determined using the basophil activation test (BAT). Upon challenge with a specific allergen, basophils not only secrete quantifiable bioactive mediators, including cytokines and histamine, but also upregulate the expression of different markers that can be detected efficiently by flow cytometry using specific monoclonal antibodies. The BAT is the flow-assisted analysis of in vitro activated basophils, which relies upon quantification of alterations in the surface expression of particular basophil activation markers [22–25]. We hypothesized that the BAT would be useful to estimate IgE-mediated allergy caused by Tg-rice seeds without oral ingestion.

The aim of this study was to examine the possibility of IgE-mediated allergy caused by Tg-rice seeds by evaluating CD203c expression on basophils of patients with JC pollinosis after in vitro stimulation with Tg-rice seed extract.

## Methods

### Subjects

This prospective study conformed to the standards of the Declaration of Helsinki and was approved by the Ethics Committee of the Jikei University School of Medicine for Biomedical Research [identification (ID): 28-265 (8508), 28-360 (8603)]. The subjects were patients with JC pollinosis who received a medical examination at the Department of Otorhinolaryngology, Jikei University School of Medicine, Tokyo, Japan and met the inclusion criteria: male or female, an age of more than 20 years, JC-specific IgE levels (ImmunoCAP) of class 2 or higher, and the presence of symptoms of JC pollinosis (sneezing and nasal itch, watery rhinorrhea, and nasal blockade) from February to April. After obtaining informed consent, whole blood samples were collected from 29 patients with JC pollinosis on the basis of the above criteria.

### Allergen extraction from the protein body powder of Tg-rice seeds

Tg-rice seeds deposit the deconstructed Cry j 1 and Cry j 2 in ER-derived protein bodies in the endosperm [15]. The protein bodies were isolated from Tg-rice seeds (Ozeki Corporation, Nishinomiya, Japan) and modified to make them powdery. Soluble allergens were extracted from powdered protein bodies as follows. First, the powdered protein bodies were dissolved in phosphate-buffered saline (PBS) at a 1:150 ratio (w/v), and the mixture was sonicated on ice. Thereafter, the mixture was centrifuged at 5800×*g* for 10 min at 4 °C, and the supernatant was collected. The supernatant was then dialyzed in PBS, concentrated tenfold using an Amicon Ultra-15 Centrifugal Filter Unit (Merck Millipore, Co. Cork, Ireland), and sterilized through a 0.22-µm Sterile Millex Filter Unit (Merck Millipore, Co. Cork, Ireland) to produce a filtered- and concentrated-Tg-rice seed extract. Extraction from the protein body powder of non-transgenic wild-type rice seeds (WT-rice seeds) was performed in the same manner as extraction from the protein body powder of Tg-rice seeds.

### Determination of the allergen concentration for the basophil activation test by using Allergen-specific lymphocyte stimulation test

Allergens examined in the BAT were Tg-rice seed extract, WT-rice seed extract, and Cry j 1 and Cry j 2, which were allergens contained in conventional SCIT and SLIT for JC pollinosis. Cry j 1 and Cry j 2 were purchased from Hayashibara Biochemical Laboratories (Okayama, Japan). WT-rice seed extract, and a mixture of Cry j 1 and Cry j 2 were compared with Tg-rice seed extract in terms of basophil activation. The most appropriate concentrations of WT- or Tg-rice seed extract, and a mixture of Cry j 1 and Cry j 2, to be used in the BAT were determined based on allergen-specific lymphocyte stimulation test. Several dilutions of WT- or Tg-rice seed extract, and a mixture of Cry j 1 and Cry j 2 were added to peripheral blood mononuclear cells (PBMCs) separated from blood samples from the first three patients with JC pollinosis among the 29 subjects. Allergen-specific lymphocyte proliferative responses to those allergens were determined using an in vitro radioactively labeled thymidine incorporation assay. Gibco Roswell Park Memorial Institute (RPMI)-1640 medium (Thermo Fisher Scientific) supplemented with 5% human AB blood type serum was used to suspend the PBMCs. Briefly, 5 × 10^5^ PBMCs were seeded into each well of a 96-Well Microplate (Nunc Microwell 96-well Microplates; Thermo Fisher Scientific.) and cultured with 1:40, 1:120, and 1:360 dilution of WT- or Tg-rice seed extract, and 0.14, 0.42, and 1.25 µg/mL of Cry j 1 and Cry j 2 in 5% CO_2_ for 72 h at 37 °C. The ratio of Cry j 1 to Cry j 2 used in the mixture was 1:1 at concentrations of 0.14, 0.42, and 1.25 µg/mL. Each well was then pulsed with 0.5 µCi of ^3^H-thymidine (American Radiolabeled Chemicals, Saint Louis, MO, USA), and the cells were harvested 16 h later using a cell harvester (Skatron Micro96 Cell Harvester; Skatron, Newmarket, UK). The level of ^3^H-thymidine incorporated by the cells was determined by measuring the radioactivity using a liquid scintillation counter (LSC-6000; ALOKA). The results were expressed as the stimulation index (SI), which was calculated as follows: mean counts per minute in the presence of the antigen divided by mean counts per minute in the absence of the antigen. An SI value greater than twice the background level was considered to indicate a positive response.

### The basophil activation test

A commercially available test system; Allergenicity Kit (Beckman Coulter) was used for the quantitative determination of basophil activation by Tg-rice seed extract. Whole blood samples from 29 patients with JC pollinosis were drawn into tubes containing heparin. The BAT was performed according to the instruction manual of Allergenicity Kit, within 4 h after blood sampling. Heparin-anticoagulated peripheral blood aliquots (100 µL) stained with 20 µL of a mixture of monoclonal antibodies (CRTH2-FITC, CD203c-PE, and CD3-PC7) were stimulated with 20 µL of each allergen (Tg-rice seed extract, WT-rice seed extract, and a mixture of Cry j 1 and Cry j 2) at 37 °C for 15 min. PBS and anti-IgE antibody (10 µg/mL) were used as negative and positive controls, respectively. Basophils express the IgE high affinity receptor, FcεRI. Anti-IgE antibody recognizes IgE bound to the receptor and consequently induces basophil activation. After incubation, the reaction was stopped using Stop solution. Erythrocytes were lysed using Lysing solution and white blood cells were fixed for 10 min at 25 °C. After centrifugation (5 min, 200×*g*), 3 mL of PBS was added to the cell pellets for washing. The cell pellet was resuspended in 0.5 mL of PBS with 0.1% formaldehyde after a second centrifugation. Basophils were detected in the low side scatter/CRTH2 positive/CD3 negative leukocyte population using a flow cytometer (MACSQuant; Miltenyi Biotec). The threshold for CD203c-positivity was set at less than 5% of activated cells in the negative control. The upper region of the threshold defines CD203c-positive activated basophils and the percentage of those cells was measured by the flow cytometer.

### Statistical analysis

Comparisons between two groups (Tg-rice seed extract vs. Cry j 1 and Cry j 2, Tg-rice seed extract vs. WT-rice seed extract) were performed using paired *t* test to determine the significance of the differences. Analysis was performed using the GraphPad Prism software (version 6.07). All tests were two-tailed and *P* values of less than 0.05 were considered significant.

## Results

### Subjects

The characteristics of all 29 subjects are shown in Table 1. The severity of JC pollinosis (most severe, severe, moderate, and mild symptoms) were determined based on paroxysmal sneezing or rhinorrhea, and nasal blockage according to Japanese guidelines for allergic rhinitis 2017 [3].

**Table 1:** The characteristics of 29 subjects

Factor	(n = 29)
Sex	
Male	15
Female	14
Age, years	42.8 ± 13.7 (21–68)
The severity of JC pollinosis	
Most severe	5 (17.2)
Severe	12 (41.4)
Moderate	9 (31.0)
Mild	3 (10.3)
JC pollen-specific IgE	
Class 2 (0.70–3.49 UA/mL)	12 (41.4)
Class 3 (3.50–17.49 UA/mL)	10 (34.5)
Class 4 (17.50–49.99 UA/mL)	4 (13.8)
Class 5 (50.00–99.9 UA/mL)	2 (6.9)
Class 6 (more than 100 UA/mL)	1 (3.4)

Values represent n (%) or mean ± SD (min.–max.)

### Allergen concentration in the basophil activation test determined by allergen-specific lymphocyte stimulation test

**Fig. 1:**
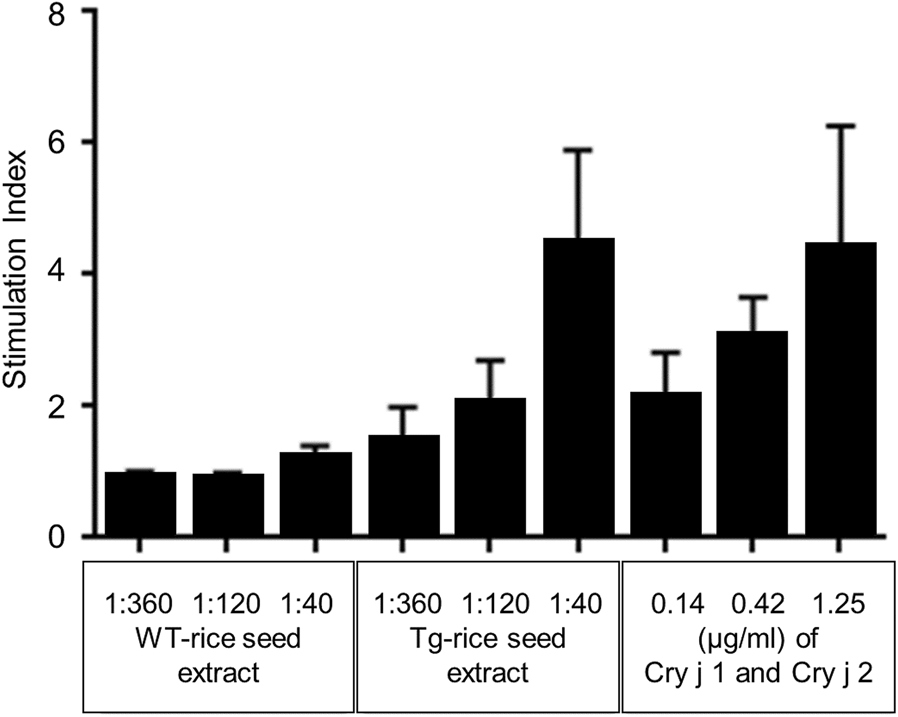
Allergen-specific lymphocyte stimulation test to determine the allergen concentration in the basophil activation test. Several dilutions of WT- or Tg-rice seed extract, and Cry j 1 and Cry j 2 were added to PBMCs separated from blood samples from the first three patients with JC pollinosis. Allergen-specific lymphocyte proliferative responses to those allergens were determined using an in vitro ^3^H-thymidine incorporation assay. The proliferation of allergen-specific lymphocytes following stimulation with 1:40 dilution of Tg-rice seed extract was almost equivalent to the proliferation following stimulation with the mixture consisting of Cry j 1 and Cry j 2 (both at 1.25 µg/mL)

The ideal concentrations of allergens to be used in the BAT were determined using allergen-specific lymphocyte stimulation test with blood samples from the first three patients with JC pollinosis. The proliferation of allergen-specific lymphocytes following stimulation with 1:40 dilution of Tg-rice seed extract was more than twice as much as that induced by 1:40 dilution of WT-rice seed extract, and it was almost equivalent to the proliferation following stimulation with the mixture consisting of Cry j 1 and Cry j 2 (both at 1.25 µg/mL) (Fig. 1). Consequently, 1:40 dilution of Tg-rice seed extract, 1:40 dilution of WT-rice seed extract, and the mixture consisting of Cry j 1 and Cry j 2 (both at 1.25 µg/mL) were adopted for use in the BAT. The proliferative response of allergen-specific lymphocytes cultured with Tg-rice seed extract compared to that induced by WT-rice seed extract also showed that antigenicity was preserved in Tg-rice seeds.

### The basophil activation test in 29 patients with JC pollinosis

**Fig. 2:**
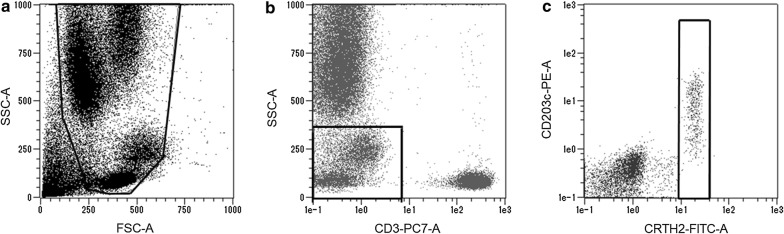
Detected basophils by flow cytometric analysis. Basophils were detected by the flow cytometer as follows. **a** Leukocyte population on forward scatter (FSC) versus side scatter (SSC) dot plot was surrounded, **b** the leukocyte population was spread on CD3-PC7 versus SSC dot plot, and CD3 negative lymphocytes and monocytes were enclosed, **c** the CD3 negative lymphocytes and monocytes were spread on CRTH2-FITC versus CD203c-PE dot plot, and the cluster of CRTH2 positive/CD3 negative leukocyte was identified as basophils

**Fig. 3:**
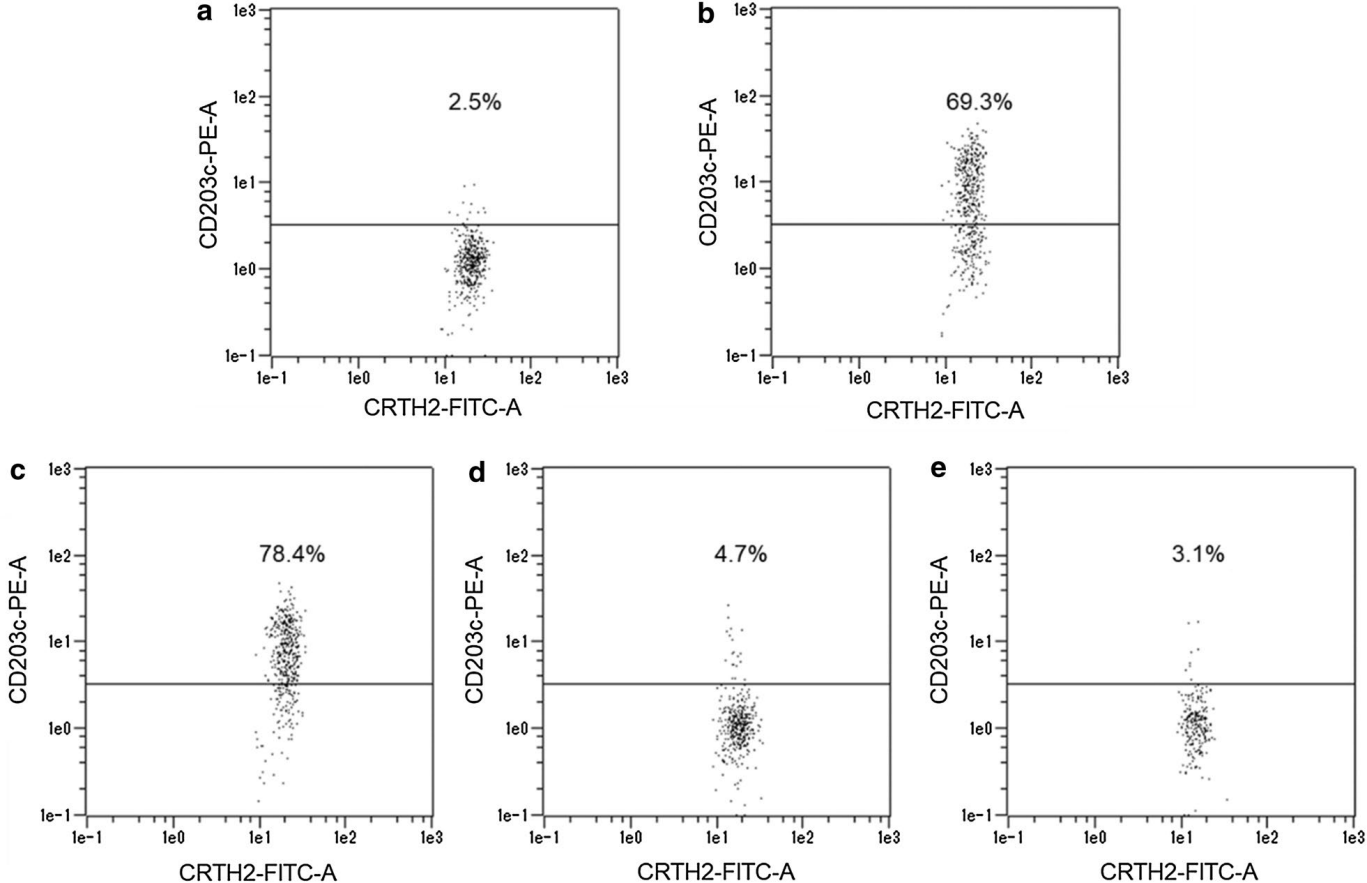
Measurement of the percentage of CD203c-positive activated basophils. One example of the basophil activation test in this study. Allergens examined in the basophil activation test were **a** PBS (negative control), **b** anti-IgE antibody (positive control), **c** Cry j 1 and Cry j 2 (allergen components of the conventional immunotherapy), **d** WT-rice seed extract, and **e** Tg-rice seed extract. The threshold for CD203c-positivity was set at less than 5% of activated cells in the negative control. The upper region of the threshold defines CD203c-positive activated basophils and the percentage of those cells was measured by the flow cytometer

**Fig. 4:**
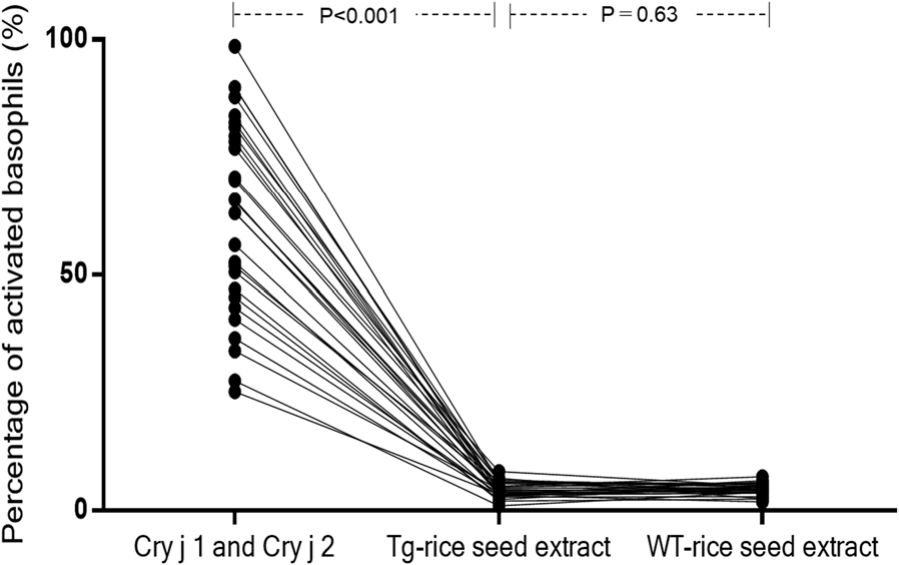
The percentage of activated basophils in 29 patients with JC pollinosis. The percentage of CD203c-positive activated basophils after the stimulation with Tg-rice seed extract was 4.5 ± 1.6% compared to 62.9 ± 20.2% after the stimulation with the mixture of Cry j 1 and Cry j 2, and this difference was significant (difference 58.4%, P < 0.001, 95% confidence interval 51.0–65.9%). There was no significant difference between Tg-rice seed extract (4.5 ± 1.6%) and WT-rice seed extract (4.6 ± 1.2%) (difference − 0.2%, P = 0.63, 95% confidence interval − 0.8% to 0.5%)

Basophil activation was evaluated by the percentage of basophils expressing the defined activation marker, CD203c. Basophils were detected in the gate for low side scatter/CRTH2 positive/CD3 negative leukocytes using the flow cytometer as follows. First, leukocyte population on forward scatter (FSC) versus side scatter (SSC) dot plot was surrounded (Fig. 2a). The leukocyte population was spread on CD3-PC7 versus SSC dot plot, and CD3 negative lymphocytes and monocytes were enclosed (Fig. 2b). The CD3 negative lymphocytes and monocytes were spread on CRTH2-FITC versus CD203c-PE dot plot, and the cluster of CRTH2 positive/CD3 negative leukocytes was identified as basophils (Fig. 2c). The threshold for CD203c-positivity was set at less than 5% of activated cells in the negative control. The upper region of the threshold defines CD203c-positive activated basophils and the percentage of those cells was measured by the flow cytometer as shown in Fig. 3. The percentage of activated basophils for the 29 patients with JC pollinosis after anti-IgE antibody stimulation was 55.3 ± 22.1% (mean ± SD), and the percentage of activated basophils after PBS stimulation was 3.7 ± 0.8%. The percentage of activated basophils after the stimulation with Tg-rice seed extract was 4.5 ± 1.6% compared to 62.9 ± 20.2% after the stimulation with the mixture of Cry j 1 and Cry j 2, and this difference was significant (difference 58.4%, P < 0.001, 95% confidence interval 51.0–65.9%) (Fig. 4). In contrast, there was no significant difference between Tg-rice seed extract (4.5 ± 1.6%) and WT-rice seed extract (4.6 ± 1.2%) in terms of the percentage of activated basophils (difference − 0.2%, P = 0.63, 95% confidence interval − 0.8% to 0.5%) (Fig. 4).

## Discussion

In the present study, the proliferative response of allergen-specific lymphocytes cultured with Tg-rice seed extract compared to that induced by WT-rice seed extract suggested that the allergen could be extracted from the protein body powder of Tg-rice seeds, as mentioned in Methods. Antigenicity of allergenic extracts from Tg-rice seed powder by the same extraction method had already been verified in mouse models by our research group. Allergen-specific lymphocytes in Cry j 1- or Cry j 2-immunized mice proliferated on treatment with Tg-rice seed extract, but not WT-rice seed extract. Moreover, T-cell lines established from the spleen cells of Cry j 1- or Cry j 2-immunized mice resulted in a proliferative response to Tg-rice seed extract, but not to WT-rice seed extract (Takaishi et al. unpublished). Tg-rice seed extracts certainly retained Cry j 1- and Cry j 2-specific antigenicity.

The basophil activation test (BAT) has been applied to investigate IgE-mediated allergy caused by classical inhalant allergens, food, *Hevea* latex, hymenoptera venoms, and drugs [23, 26–30]. Oral food challenge is one of the methods used to identify the causative food in the diagnosis of food allergy [31]. However, oral ingestion of the examined food might cause acute allergic reactions, including an anaphylactic reaction. Therefore, oral food challenge needs to be performed in a supervised environment with the facilities and expertise to treat allergic reactions [26]. In recent years, the BAT has been used as a new diagnostic test for food allergy. Acute allergic reactions to a food extract can be observed in vitro without oral ingestion using the BAT. A general agreement was found between the results of the BAT and the outcome of oral food challenge in several studies [32, 33]. The BAT confers a high degree of certainty in confirming the diagnosis of food allergy [26]. The diagnostic utility of the BAT is allergen-specific and can be validated for different allergens; therefore, we believed that the BAT could be applied to evaluate the possibility of IgE-mediated allergy caused by Tg-rice seeds.

In the present study, the BAT showed that the percentage of activated basophils after the stimulation with Tg-rice seed extract was significantly lower compared with that induced by the mixture of Cry j 1 and Cry j 2. Tg-rice seed extract caused very little activation of basophils in the patients with JC pollinosis compared with that induced by the mixture of Cry j 1 and Cry j 2. Furthermore, there was no significant difference between Tg-rice seed extract and WT-rice seed extract in terms of the percentage of activated basophils. These results showed that Tg-rice seeds would be much safer than conventional immunotherapies using crude antigens including Cry j 1 and Cry j 2 and might be equally safe as WT-rice seeds in terms of the risk of IgE-mediated allergic reactions.

Recombinant DNA technology produced specific and safe vaccines with reduced IgE-binding. There are many dropouts during the processes of SCIT and SLIT, since both SCIT and SLIT for JC pollinosis require the patients to receive regular outpatient treatment, at least once a month, over 3 years. The reduced risk of IgE-mediated allergy may allow high-dose administration of the modified antigens and therefore, a shortening of the duration of immunotherapy. Oral immunotherapy with the transgenic rice might improve treatment adherence because rice is a principal food of the Japanese. The introduction of the transgenic rice to immunotherapy could circumvent the problems concerning adherence, dropouts, and adverse events. Moreover, oral immunotherapy with the transgenic rice may contribute to health economics, since oral intake of rice would require neither food processing nor allergen component extraction from rice seeds. There is even a possibility that production of Tg-rice seeds would become a new industry in agriculture. Besides being a new type of immunotherapy for after the onset of symptoms, consumption of Tg-rice seeds via the oral route may be preventive strategy, acting prior to the development of JC pollinosis, if successful mucosal immune tolerance could be obtained. Tg-rice seeds may be provided as a rice-based allergy vaccine, with reduced IgE-binding, against JC pollinosis.

There is, however, a limitation to this study. Although intake of drugs, particularly glucocorticosteroids and immunosuppressive drugs, might interfere with the BAT, the exclusion criteria concerning the medication are not yet established. However, as a positive-control stimulus, anti-IgE antibody was applied to the blood sample of every subject. Amongst the 29 subjects, there was no negative result from anti-IgE antibody challenge. Approximately 5–10% of the individuals tested fail to upregulate CD63 and CD203c with regard to IgE-mediated basophil activation [23, 24]. There was no “non-responder” among the 29 subjects. Had there been any, it would have been difficult to distinguish “non-responder” from the subjects who are under immunosuppression due to medication.

## Conclusions

In conclusion, these results will contribute to the safety of Tg-rice seeds in terms of IgE-mediated allergy. We hope that this study forms a bridge to initiate a clinical trial of Tg-rice seeds.

## Abbreviations

JC: Japanese cedar
SCIT: subcutaneous immunotherapy
SLIT: sublingual immunotherapy
IgE: immunoglobulin E
Tg-rice seeds: the transgenic rice seeds expressing whole T-cell epitopes of the major Japanese cedar pollen allergens Cry j 1 and Cry j 2
BAT: basophil activation test
PBS: phosphate-buffered saline
WT-rice seeds: non-transgenic wild-type rice seeds
PBMCs: peripheral blood mononuclear cells
